# The effect of varying inhaled oxygen concentrations of high-flow nasal cannula oxygen therapy during gastroscopy with propofol sedation in elderly patients: a randomized controlled study

**DOI:** 10.1186/s12871-022-01879-z

**Published:** 2022-11-02

**Authors:** Wenwen Zhang, Hailing Yin, Yajie Xu, Zhaojing Fang, Wanling Wang, Chen Zhang, Hongwei Shi, Xiaoliang Wang

**Affiliations:** Department of Anesthesiology, Nanjing First Hospital, Nanjing Medical University, Nanjing, China

**Keywords:** High-flow nasal cannula, Inhaled oxygen concentrations, Gastroscopy, Sedation, Elderly patients, Hypoxia

## Abstract

**Background:**

Despite evidence that high-flow nasal cannula oxygen therapy (HFNC) promotes oxygenation, its application in sedated gastroscopy in elderly patients has received little attention. This study investigated the effect of different inhaled oxygen concentrations (FiO_2_) of HFNC during sedated gastroscopy in elderly patients.

**Methods:**

In a prospective randomized single-blinded study, 369 outpatients undergoing regular gastroscopy with propofol sedation delivered by an anesthesiologist were randomly divided into three groups (*n* = 123): nasal cannula oxygen group (Group C), 100% FiO_2_ of HFNC group (Group H100), and 50% FiO_2_ of HFNC (Group H50). The primary endpoint in this study was the incidence of hypoxia events with pulse oxygen saturation (SpO_2_) ≤ 92%. The secondary endpoints included the incidence of other varying degrees of hypoxia and adverse events associated with ventilation and hypoxia.

**Results:**

The incidence of hypoxia, paradoxical response, choking, jaw lift, and mask ventilation was lower in both Group H100 and Group H50 than in Group C (*P* < 0.05). Compared with Group H100, Group H50 showed no significant differences in the incidence of hypoxia, jaw lift and mask ventilation, paradoxical response, or choking (*P* > 0.05). No patients were mechanically ventilated with endotracheal intubation or found to have complications from HFNC.

**Conclusion:**

HFNC prevented hypoxia during gastroscopy with propofol in elderly patients, and there was no significant difference in the incidence of hypoxia when FiO_2_ was 50% or 100%.

**Trial registration:**

This single-blind, prospective, randomized controlled trial was approved by the Ethics Committee of Nanjing First Hospital (KY20201102-04) and registered in the China Clinical Trial Center (20/10/2021, ChiCTR2100052144) before patients enrollment. All patients signed an informed consent form.

## Background

Gastric cancer is the most prevalent malignant tumour of the digestive system, and its crude mortality, incidence, and prevalence in 2017 were more significant than those in 1990 in China [1–3]. An increasing number of elderly patients undergo gastroscopy while sedated, with preserved spontaneous respiration, resulting in a high level of patient comfort. However, hypoxia occurs in approximately 1.5–70% of cases during the peri-examination period, of which 36% are associated with apnea and 30% with abnormal ventilation [4]. Severe adverse events occurr more frequently in patients over 65 years of age who are at greater risk of hypoxia during sedated endoscopy [5, 6]. Regarding complex endoscopies, Wani et al. found that age was an independent predictor of adverse events related to sedation [7]. Sedation-related hypoxia events are more frequent in elderly patients because of physiopathological changes such as decreased active alveolar substance, decreased lung conformance, and altered parenchymal function. Therefore, it is essential to find a proper ventilation strategy for elderly patients in gastroscopy with sedation.

High-flow nasal cannula oxygen therapy (HFNC) is a recently developed approach that uses a particular nasal cannula to provide very high flow (up to 60 L/min) heated and humidified gas with adjustable temperatures (31 – 37 °C) and oxygen concentrations (21 – 100%) [8]. By supplying high-flow oxygen, HFNC can rapidly wash away carbon dioxide (CO_2_) in the nasopharyngeal cavity and create positive airway pressure (3–7 cmH_2_O), thereby increasing end-expiratory lung capacity. It is possible that high-flow oxygen delayed the recognition of severe hypoventilation/apnea. However, according to Michael et al., the incidence of hypercarbia among patients undergoing advanced esophagogastroduodenoscopy was not significantly different between HFNC and standard nasal cannula oxygen. HFNC has been demonstrated to be safe for sedated gastroscopy due to the higher flow and inhaled oxygen concentrations (FiO_2_ ) [9].

For hypoxemia, high levels of FiO_2_ are commonly inhaled, but excessive exposure to hyperoxia is negative [10]. High oxygen concentrations increase pulmonary oxidative stress, reduce alveolar surfactant levels, and increase microcirculation vasoconstriction, further damaging the lungs [11, 12]. Moreover, high oxygen concentrations can cause absorption pulmonary atelectasis in small closed airways due to oxygen absorption, increase intrapulmonary shunt rates, and influence pulmonary ventilation function [13, 14]. Accelerated rehabilitation surgery recommends reducing the FiO_2_ and avoiding prolonged inhalation of oxygen concentrations over 80% while maintaining a normal arterial partial pressure and oxygen saturation [15]. Therefore, this study was designed to compare different FiO_2_ values of HFNC with oxygen via nasal cannula to investigate whether different FiO_2_ values in of HFNC can reduce the incidence of hypoxia in elderly patients undergoing sedated gastroscopy.

## Methods

### Ethics and registration

This single-blind, prospective, randomized controlled trial was approved by the Ethics Committee of Nanjing First Hospital (KY20201102-04) and registered in the China Clinical Trial Center (20/10/2021, ChiCTR2100052144) before patients enrollment. All patients signed an informed consent form.

### Study participants

Patients scheduled for propofol-sedated gastroscopy were enrolled in this study. The inclusion criteria were as follows: (1) ≥ 65 years old; (2) ASA classification of I - II; and (3) BMI < 30 kg/m^2^. The following were the exclusion criteria: (1) emergency endoscopy; (2) upper gastrointestinal obstruction and impaired gastric emptying; (3) coagulation disorders or upper respiratory bleeding, severe cardiovascular, pulmonary, liver, or kidney disease; (4) infections or tumours of the mouth, nose, or pharynx; (5) a history of difficult intubation, severe sleep apnea syndrome [respiratory/pause hypoventilation index (AHI) > 40]; (6) allergy to propofol, eggs, soy or egg whites; and (7) unaccompanied or unattended individuals.

### Randomization and blinding

The research equipment included the HFNC (AIRVO 2 provided by Fisher & Paykel, Panmure, New Zealand) and a regular nasal cannula.

Using computer randomization software (SPSS 24.0), each patient was randomly categorized into a nasal cannula oxygen group (Group C), 100% FiO_2_ in HFNC group (Group H100), and 50% FiO_2_ in HFNC group (Group H50) in a 1:1:1 ratio. The grouping results were secured in a sealed opaque envelope, which was opened only by the researchers before anesthesia induction. Blinded members of the research team conducted postoperative interviews with the patients. A member of the study team worked on registering and assigning patients, who were unaware of the randomization grouping, and the anesthesiologists involved in the study were trained and qualified in the use of HFNC.

### Interventions and anesthesia

The patients fasted for 8 h and ceased drinking for 2 h before the gastrointestinal endoscopy procedure and took no more than 50 ml of mucosal cleanser 30 min before the procedure. The specific preoperative preparation requirements were in accordance with the relevant guidelines for gastrointestinal endoscopic procedures.

Riphaus demonstrated that bolus and continuous propofol infusions provide similar good controllability of propofol sedation. Therefore, an IV push technique was adopted rather than an infusion pump [16]. Following arrival to the operating room, patients were routinely monitored with electrocardiography, respiration, blood pressure, and SpO_2_. Then, they were instructed to lie on their left side. In Group C, 8 L/min pure oxygen was used via a nasal cannula for calm breathing, while 30 L/min oxygen was used via HFNC in both Groups H100 and H50. The FiO_2_ values of Groups H100 and H50 were 100% and 50%, respectively. Each group underwent a minute of calm breathing with adequate denitrogenation. The initial load of propofol was slowly administered intravenously at a dose of 1.5–2.5 mg/kg. After the patients’ Ramsay sedation score reached 4, the flow was increased to 60 L/min in Groups H100 and H50 while keeping FiO_2_ constant. The gastroenterologist began the endoscopic procedure. If the consultation time was slightly longer or the stimulation of the operation was stronger, additional propofol 0.2–0.5 mg/kg was injected intravenously. An extra dose based on the patient’s signs, such as deepening respiration, increased heart rate, and even paradoxical response, was used and included in the record of the propofol dosage. No benzodiazepines or opioids were used. When treating the patient, maintaining a good level of anesthesia and sedation was essential to ensuring unconsciousness.

During the peri-examination period, hypoxia (SpO_2_ ≤ 92%) was treated via the following protocols: (1) stimulate the patient; (2) stop the medication; (3) increase the oxygen flow rate from 8 to 10 L/min in Group C; adjust the FiO_2_ to 100% in Group H50; (4) open the airway by lifting the jaw; (5) exit the gastroscope and parallel mask ventilation; and (6) perform endotracheal intubation for mechanical ventilation. During the examination, ephedrine 5–10 mg was administered intravenously if hypotension was found (systolic pressure below 80 mmHg for more than 1 min); atropine 0.25–0.50 mg was administered if the heart rate was less than 50 beats per minute. If necessary, the drug administration was repeated.

### Outcomes and data collection

The incidence of hypoxia during the peri-examination period (SpO_2_ ≤ 92%) was the primary endpoint. Kelly AM et al. suggested that SpO_2_ ≤ 92% was a valid indicator for screening for systemic hypoxia, although hypoxia is defined as SpO_2_ < 90%. Based on previous research [17, 18], the inclusion of elderly patients and 50% FiO_2_ in Groups C and H50, our study defined SpO_2_ ≤ 92% as hypoxia [19].

The following were secondary endpoints: (1) hypoxia-related indicators: incidence of SpO_2_ < 90%; incidence of severe hypoxia (SpO_2_ ≤ 85%); incidence of prolonged hypoxia (SpO_2_ ≤ 92% for 1 min); SpO_2_ 1 min after anesthesia induction; (2) interventions used to treat hypoxia events; (3) patient’s overall condition and airway assessment; (4) general information about gastroscopy(duration, dose of propofol and wake-up time); (5) adverse events related to HFNC ventilation at 5 and 30 min postoperatively(airway injury or any barotrauma including pneumothorax, subcutaneous emphysema, etc.); and (6) any sedation-related adverse events(paradoxical response, nausea/vomit, reflux, airway obstruction, or choking).

### Sample size

The primary endpoint of this study was the incidence of SpO_2_ ≤ 92% during the peri-examination period. According to the preliminary test results, the incidence of SpO_2_ ≤ 92% during the peri-examination period was 33%, 15%, and 20% in Groups C, H100, and H50, respectively. According to PASS 11.0 (NCSS, LLC., Kaysville, UT, USA), with an error of 0.05 (two-tailed) and a power of 0.80, 294 patients were needed. Due to attrition, a total of 369 patients was finally identified (123 patients in each group).

### Statistical analysis

SPSS software (version 24.0; SPSS, Inc., Chicago, IL, USA) was used for the statistical analysis. We performed the Shapiro-Wilk test and Levene’s test on all continuous variables. According to the test results, the data are expressed as the means ± standard deviations or medians [interquartile ranges]. The normally distributed measurement data (dose of propofol) were compared between groups by one-way ANOVA, and the LSD method was further used for multiple comparison. Measurement data with skewed distribution (duration, wake-up time, median 1 min after induction of anesthesia SpO_2_) were compared between groups using the rank sum test, and Dunn’s method was used for multiple comparisons. We compared the incidence of hypoxia and sedation-related adverse events between groups with a chi-square test or Fisher’s exact test, and the P value of the multiple comparison was corrected by the Bonferroni method. Statistical significance was set at *P* < 0.05.

## Results

**Fig. 1:**
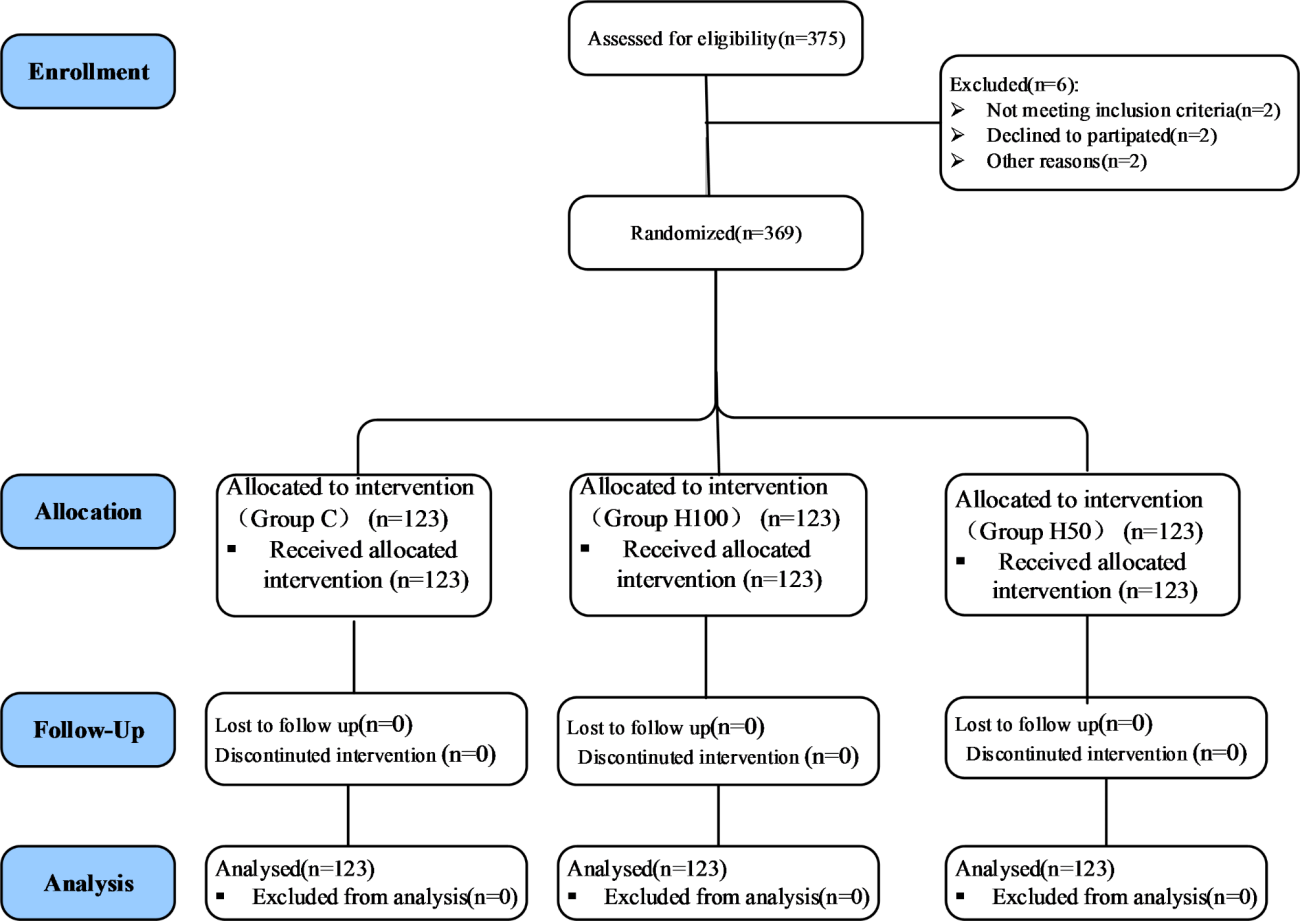
CONSORT Flow chart

From October 2021 to February 2022, 375 patients were enrolled, of whom six were excluded (3 patients did not meet the inclusion criteria, and three declined to participate). Finally, 369 patients were randomized into three groups. After patient enrollment, we had no attrition/withdrawal owing to severe hypoxemia, resuscitation, or protocol violations (Fig. 1).

There were no significant differences between the general data and airway assessment indicators of the three groups (*P* > 0.05) (Table 1). The same was true for sedated gastroscopy time, propofol dosage, and wake-up time among the three groups (*P* > 0.05) (Table 2).

**Table 1:** General characteristics of patients

Characteristics	Group C (n = 123)	Group H100 (n = 123)	Group H50 (n = 123)	P value
Age, yr	70(68, 72)	69(67, 71)	70(67, 72)	0.062
Sex male/female (n)	47/76	52/71	49/74	0.807
BMI, ( kg/m^2^)	22.8 ± 2.9	22.5 ± 2.9	22.4 ± 2.5	0.538
ASA grade (I/II)	37/86	34/89	38/85	0.844
Mallampati class ( I/II/III/IV)	108/15/0/0	105/18/0/0	103/20/0/0	0.658
Mouth opening (1/2/3)	0/8/115	0/10/113	0/11/112	0.770
Thyromental distance(I/II/III)	107/16/0	103/20/0	106/17/0	0.751
Baseline SpO_2_	98(97, 99)	98(97, 99)	98(97, 98)	0.101

Values were presented as mean ± SD, a number of patients, number (%), or median (Q1, Q3). BMI: Body mass index ASA grade: American Society of Anesthesiologists physical status. Mouth opening (1)(2)(3): 1 = 1 finger, 2 = 2 fingers, 3 = 3 fingers. Thyromental distance (I)(II)(III): I > 6.5 cm, II 6–6.5 cm, III < 6 cm. Group C: nasal cannula oxygen group, Group H100: 100% of FiO_2_ from HFNC group, Group H50: 50% of FiO_2_ from HFNC group

**Table 2:** Data of gastroscopy procedure

	Group C (n = 123)	Group H100 (n = 123)	Group H50 (n = 123)	P value
Duration, min	9 (8, 11)	9 (8, 11)	9 (8, 10)	0.177
Dose of propofol, mg	139.6 ± 22.3	143.2 ± 22.1	142.7 ± 18.5	0.068
Wake-up time, min	3 (3, 4)	3 (2, 4)	4 (3, 4)	0.056

Values were presented as mean ± SD and median (Q1, Q3)

When hypoxia was defined as SpO_2_ ≤ 92%, the incidence rates were 30.1%, 12.2%, and 14.6% in Groups C, H100, and H50, respectively. The incidence of hypoxia, the rate of jaw lift, and the use of mask ventilation for hypoxia adverse events were significantly lower in Group H100 and Group H50 than in Group C (*P* < 0.05). However, no significant differences were seen in the incidence of hypoxia, the rate of jaw lift, or the use of mask ventilation for hypoxia adverse events between Group H50 and Group H100 (*P* > 0.05) (Table 3).

**Table 3:** Respiratory-related adverse events and intervention

	Group C n = 123	Group H100 n = 123	Group H50 n = 123	P value
Hypoxia	37(30.1)	15(12.2)^*^	18(14.6)^*^	0.001
SpO_2_ < 90%	27(22.7)	7(5.7)^*^	9(7.3)^*^	*P* < 0.001
Severe hypoxia	9(7.3)	3(2.4)^*^	2(1.6)^*^	0.041
Prolonged hypoxia	10(8.1)	2(1.6)	2(1.6)	0.009
Median 1 min after induction of anesthesia SpO_2_	99(98, 100)	100(99, 100)	100(99, 100)	0.063
Jaw lift	36(29.3)	12(9.8)^*^	11(8.9)^*^	*P* < 0.001
Mask ventilation	15(12.2)	0(0)^*^	0(0)^*^	*P* < 0.001
Mechanical ventilation for endotracheal intubation	0(0)	0(0)	0(0)	1.000

Values were presented as a number of patients, number (%), or median (Q1, Q3). ^*^Hypoxia、^*^SpO_2_<90%, ^*^Severe hypoxia, ^*^Jaw lift, and ^*^Mask ventilation were significantly lower in groups H100 and H50, compared to group C

In this study, no patients in any group underwent endotracheal intubation for mechanical ventilation. Compared with Group C, the incidence of paradoxical response and choking was reduced in Group H100 and Group H50 (*P* < 0.05), while no significant difference was seen in the incidence of paradoxical response and choking between Group H50 and Group H100 (*P* > 0.05) ( Table 4). Adverse events related to HFNC ventilation were recorded at 5 and 30 min after patients awakened, and no patients were found to have pneumatic injuries or airway injuries.

**Table 4:** Sedation-related adverse events

	Group C n = 123	Group H100 n = 123	Group H50 n = 123	P value
Paradoxical response	14(11.4)	2(1.6)^*^	2(1.6)^*^	*P* < 0.001
Nausea/vomit	0(0)	0(0)	0(0)	1.000
Reflux	0(0)	0(0)	0(0)	1.000
Airway obstruction	0(0)	0(0)	0(0)	1.000
Choking	14(11.4)	2(1.6)^*^	2(1.6)^*^	*P* < 0.001

Values were presented as a number of patients, number (%), or median (Q1, Q3). Paradoxical response: Patients displayed unpredictable movement, overexcitement, and delirium after sedation with propofol. Airway obstruction: Patients had glossoptosis, excessive oropharynx secretion, laryngeal spasm, or bronchospasm. ^*^Paradoxical response and ^*^Choking were significantly lower in groups H100 and H50, compared to group C

## Discussion

Compared to nasal cannula oxygenation, HFNC decrease the incidence of SpO_2_ ≤ 92% from 30.1 to 12.2% and 14.6% during sedated gastroscopy of elderly patients in our study. Mild airway obstruction and incomplete respiratory depression were the leading causes of respiratory depression during sedated gastroscopy. Compared to low flow oxygen, HFNC allowed the actual FiO_2_ to be closer to the set FiO_2_. Additionally, the high flow of HFNC significantly reduced nasopharyngeal resistance and flushed the nasopharyngeal cavity, allowing gas to enter the lower airway more smoothly. Parke’s research found that when HFNC was between 30 L/min and 50 L/min, every 10 L/min increase in flow rate increased the nasopharyngeal pressure by approximately 1 cmH_2_O and the mean airway pressure by 0.35 cmH_2_O in patients with open mouths. When the oxygen flow rate was 60 L/min, the calculated airway pressure was approximately 2 cmH_2_O, relieving mild airway obstruction and partial respiratory depression while keeping SpO_2_ stable [20, 21]. When the patient had a low tidal volume and frequency of ventilation, the high flow rate of 60 L/min kept a small airway opening by maintaining a low level of positive airway pressure, allowing for O_2_ and CO_2_ exchange [22, 23]. In the HFNC groups, with the stable FiO_2_, the oxygen flow was from 30 L/min to 60 L/min, and there was no significant difference in the incidence of hypoxia. The above findings demonstrated that the effects of a high flow rate on airway flushing and a low level of positive airway pressure were more effective in maintaining the oxygenation of patients.

In our study, hypoxia was not significantly different between the two groups when FiO_2_ was 100% or 50%. At present, the optimal concentration of inhaled oxygen is not consistent, but prolonged inhalation of high oxygen concentrations will cause an overreaction to oxidative stress. It is recommended that anesthesiologists use the lowest FiO_2_ possible while preserving the patient’s normal oxygen supply and reducing oxygen concentration-related lung injury whenever there is no strong evidence to support the use of high FiO_2_ [24]. For elderly patients, 50% FiO_2_ in HFNC could meet the oxygenation requirement during sedated gastroscopy. Rather than titrating hypoxia, this study compared high flow oxygen inhalation to conventional nasal cannula oxygen inhalation when FiO_2_ was 50%. Based on the results of this study, we can use titration to find the optimal concentration of inspired oxygen in the future.

Our study demonstrated the effectiveness of HFNC in preventing hypoxia during sedated gastroscopy in elderly patients, but the incidence of severe hypoxia (SpO_2_ ≤ 85%) and prolonged hypoxia (SpO_2_ ≤ 92% and lasting 1 min) were not 0. In contrast, Su Dian San could even reduce the incidence of hypoxia (75% < SpO_2_ < 90% and time < 60 s) and severe hypoxia (SpO_2_ < 75% or 75% < SpO_2_ < 90% and time > 60 s) to 0 in ASA class I - II patients [13]. The three possible explanations are as follows: (1) the population included in this study comprised elderly patients with pre-existing pathophysiological changes in respiratory function; (2) the duration of sedated gastroscopy was approximately 9 min, which was significantly longer than the 5 min in their study; and (3) the definition of hypoxia in this study was different.

In addition, when the FiO_2_ was similar to that in the nasal cannula, hypoxia was significantly reduced in the HFNC group, whereas Riccio’s study found that sedated colonoscopy did not benefit morbidly obese patients [25]. In addition to the sedation protocol, the definition of hypoxia differed, with the main difference being that they included patients with BMI > 40 kg/m^2^ for colonoscopy. Colonoscopy results in a higher rate of upper airway obstruction in patients who were morbidly obese.

The shortcomings of this study are as follows: (1) The definitions of hypoxia were different, and there were significant differences in the range and duration of SpO_2_ in the various definitions. Thus the specific differences between the incidence of hypoxia and previous studies could not be reflected by statistical data. (2) When the oxygen flow rate of HFNC was too high, it was not possible to accurately monitor end-expiratory carbon dioxide. As a result of limited hospital funds, blood carbon dioxide levels were not measured.

## Conclusion

HFNC can prevent hypoxia in elderly patients with ASA I–II status who are undergoing gastroscopy with propofol anesthesia. Whether FiO_2_ is 50% or 100% has no significant effect on the hypoxia incidence in HFNC groups.

## Data Availability

The datasets generated and/or analysed during the current study are not publicly available due to policy issues in the hospital but are available from the corresponding author on reasonable request.

## Abbreviations

HFNC: high-flow nasal cannula oxygen therapy
FiO_2_: inhaled oxygen concentrations
SpO_2_: pulse oxygen saturation
CO_2_: Carbon dioxide
